# Supplementary material for: Empirical methods for controlling false positives and estimating confidence in ChIP-Seq peaks
Source: BMC Bioinformatics. 2008 Dec 5;9:523. doi: 10.1186/1471-2105-9-523 (PMC2628906; doi:10.1186/1471-2105-9-523)
Supplement: Additional File 1 — A variety of html documents from the USeq web site detailing the available applications, their best usage, output file type descriptions, command line menus, etc. [file 1471-2105-9-523-S1.zip › USeqUserGuides/JavaDoc.zip!/JavaDoc/edu/utah/seq/data/class-use/Info.html]

Uses of Class edu.utah.seq.data.Info


|  |  |  |
| --- | --- | --- |
| **Overview** \| **Package** \| **Class** \| **Use** \| **Tree** \| **Deprecated** \| **Index** \| **Help** | |  |
| PREV   NEXT | **FRAMES**    **NO FRAMES**     **All Classes** |  |


---


## **Uses of Class edu.utah.seq.data.Info**

| Packages that use Info | |
| --- | --- |
| **edu.utah.seq.analysis** | Primary analysis applications and helper classes for working with binary PointData, xxx.bar.zip files. |
| **edu.utah.seq.data** | Helper classes for working with binary PointData, EnrichedRegions, etc. |

Uses of Info in edu.utah.seq.analysis

| Methods in edu.utah.seq.analysis with parameters of type Info | |
| --- | --- |
| `void` | `ScanSeqs.savePointAndHeatMapData(java.io.File saveDir, int scoreIndex, int scoreID, SmoothingWindow[] sm, Info info, boolean twoColor)`             Saves point and heat map bar files of a given score. |
| `void` | `ScanSeqs.saveSmoothedHeatMapData(int scoreIndex, SmoothingWindow[] sm, Info info, java.io.File dir, java.lang.String description, java.lang.String units, boolean twoColor)` |
| `void` | `ScanSeqs.saveSmoothedPointData(int scoreIndex, SmoothingWindow[] sm, Info info, java.io.File dir, java.lang.String description, java.lang.String units)` |

Uses of Info in edu.utah.seq.data

| Methods in edu.utah.seq.data that return Info | |
| --- | --- |
| `Info` | `SmoothingWindowInfo.getInfo()` |
| `Info` | `PointData.getInfo()` |
| `Info` | `InfoPoint.getInfo()` |

| Methods in edu.utah.seq.data with parameters of type Info | |
| --- | --- |
| `void` | `SmoothingWindowInfo.setInfo(Info info)` |
| `void` | `PointData.setInfo(Info info)` |
| `void` | `InfoPoint.setInfo(Info info)` |

Constructors in edu.utah.seq.data with parameters of type Info

`EnrichedRegion(SmoothingWindow window,
Info info)`

`InfoPoint(int position,
float score,
Info info)`

`SmoothingWindowInfo(SmoothingWindow[] sm,
Info info)`

---


|  |  |  |
| --- | --- | --- |
| **Overview** \| **Package** \| **Class** \| **Use** \| **Tree** \| **Deprecated** \| **Index** \| **Help** | |  |
| PREV   NEXT | **FRAMES**    **NO FRAMES**     **All Classes** |  |


---
